# EGFRvIII: the promiscuous mutation

**DOI:** 10.1038/cddiscovery.2016.49

**Published:** 2016-07-04

**Authors:** Sameer A Greenall, Terrance G Johns

**Affiliations:** 1 Centre for Cancer Research, Hudson Institute of Medical Research, Clayton, Victoria 3168, Australia; 2 Monash University, Clayton, Victoria 3800, Australia

High-grade glioma (HGG) is the most common neoplasm of the brain and is almost universally lethal.^1^ One of the most frequently altered genes in HGG is the epidermal growth factor receptor (*EGFR*) gene.^2^ The most common alteration is amplification of wild-type (wt) *EGFR*, which is observed in around half of all HGG tumors. About half of these (about one-quarter of all HGG tumors) also contain a mutation known as EGFRvIII,^2^ which is strongly tumorigenic. The signaling mechanisms underlying EGFRvIII’s tumorigenicity are not fully understood. Now, in a recent issue of *Nature Neuroscience*, Jahani-Asl *et al*.^3^ add to our knowledge of how EGFRvIII mediates its profound tumorigenicity.

Previous studies have shown that EGFRvIII is a gain-of-function mutation that arises from genomic deletion of exons 2–7.^4^ This deletion leads to a ligand-independent receptor, although the constitutive activity of this receptor is lower than that of ligand-stimulated wtEGFR.^4^ Despite this sub-optimal activity, EGFRvIII imparts a significant growth advantage to HGG cells, especially *in vivo*. Moreover, because of this low level of activation, EGFRvIII is not downregulated, unlike wtEGFR following activation by ligand. This leads to sustained signaling through EGFRvIII,^5^ which is critical to its tumorigenicity.

Nonetheless, this low level of sustained signaling through EGFRvIII is insufficient to explain its full tumorigenic activity, which promotes a diverse range of biological activities, including proliferation, survival and angiogenesis.^4^ There is some evidence of how EGFRvIII mediates its diverse range of biological effects. For example, we have shown that EGFRvIII signaling is amplified through interaction with a range of other receptor tyrosine kinases, especially c-Met.^6^ Indeed, EGFRvIII forms a heterodimer with c-Met, leading to its direct activation and enhancing its response to the c-Met ligand hepatocyte growth factor.^6^ As a consequence, the co-targeting of EGFRvIII and c-Met has synergistic anti-tumor activity in mouse xenograft models.^6^

In addition, EGFRvIII has been shown to promote HGG growth through paracrine mechanisms. Cells expressing EGFRvIII secrete large amounts of interleukin-6 and leukemia inhibitory factor.^7^ These cytokines can activate signaling through gp130 in neighboring cells. Activated gp130, in turn, activates wtEGFR, even in the absence of EGFRvIII in these cells or of EGFR ligand, thereby enhancing HGG cell growth both *in vitro* and in xenograft models.^7^

The recent study by Jahani-Asl *et al*.^3^ broadens our understanding of the tumorigenic capacity of EGFRvIII. The authors conducted global RNA sequencing (RNA-seq) on a series of HGG stem cell lines that express EGFRvIII. Using the knowledge that EGFRvIII activates the transcription factor STAT3, they simultaneously performed RNA-seq on mouse astrocytes (one of the normal cells of origin for HGG) expressing EGFRvIII in the presence or absence of *Stat3*. Finally, they also conducted chromatin immunoprecipitation sequencing on mouse astrocytes expressing both EGFRvIII and STAT3. Using this wealth of data, the authors were able to identify genes whose expression is driven specifically by the EGFRvIII–STAT3 pathway.

The primary ‘hit’ from this analysis was the receptor for the cytokine oncostatin M. This receptor, OSMR, was highly expressed in all tested HGG stem cell lines and in the EGFRvIII-expressing mouse astrocytes. Furthermore, STAT3 clearly bound to the *Osmr* gene promoter, and knockdown of *Stat3* by lentivirus-mediated RNA interference resulted in a marked reduction in the level of *Osmr* mRNA, indicating that OSMR is probably a positive feed-forward signal in the EGFRvIII–STAT3 pathway (Figure 1).

Using classical biochemical techniques such as co-immunoprecipitation and newer imaging techniques such as proximity ligation assay, the authors also showed a direct interaction between the EGFRvIII and OSMR proteins. This interaction was dependent on the phosphorylation (activation) of EGFRvIII, as pharmacological inhibition of EGFRvIII with EGFR-directed tyrosine kinase inhibitors prevented the association of EGFRvIII and OSMR.

The canonical signaling pathway for OSMR involves binding its cognate ligand (OSM), direct interaction with its co-receptor (gp130) and subsequent activation of the JAK–STAT pathway.^8^ Surprisingly, the authors show that the interaction of EGFRvIII and OSMR and the subsequent signaling of the EGFRvIII–OSMR complex is independent of gp130. They go on to demonstrate a similar direct interaction between OSMR and wtEGFR; however, the formation of this receptor complex was dependent on OSM. One important follow-up question from these studies is whether the EGFRvIII–OSMR complex also directly activates JAK signaling and, as a corollary, whether this activation is augmented by the addition of exogenous OSM. If this pathway is activated, there is a readily available path into the clinic using FDA-approved JAK inhibitors such as ruxolitinib.^9^

EGFRvIII is known to contain a free cysteine that is not present in untruncated EGFR, and we have shown that this cysteine is critical for the activation of EGFRvIII and for its ability to transactivate other receptor tyrosine kinases such as c-Met.^6^ Uncovering the role of this cysteine in the EGFRvIII–OSMR complex may provide new mechanistic insight into how this complex is formed and subsequently signals.

Importantly, the authors also analyzed two HGG databases and showed that high levels of OSMR were significantly associated with poorer survival. Consistent with this, knockdown of *OSMR* in the HGG stem cell lines reduced their tumorigenicity in an intracranial model, and knockdown of *Osmr* in EGFRvIII-expressing mouse astrocytes robustly reduced their *in vivo* tumor growth by attenuating cell proliferation. Knockdown of *Osmr* in EGFRvIII-expressing mouse astrocytes also had the surprising effect of significantly reducing EGFRvIII expression, which correlated with loss of phosphorylated STAT3. This raises the possibility that phospho-STAT3, driven by OSMR, may sustain EGFRvIII expression. This idea could be further explored in the human EGFRvIII-expressing HGG stem cell lines after *OSMR* knockdown. If replicated, the therapeutic targeting of OSMR could inhibit both EGFRvIII and STAT3 simultaneously. Taken together, these data suggest that targeting OSMR signaling in patients with EGFRvIII-positive HGG tumors would have therapeutic benefit.

This new study strongly supports the idea that EGFRvIII is a valid therapeutic target in HGG. Despite this, attempts to therapeutically target EGFR and EGFRvIII with single agents have largely failed in patients,^10^ even when the drug (e.g., gefitinib) reaches high enough concentrations within the tumor.^11^ This study raises the possibility that resistance to EGFR-targeted therapeutics could be mediated by EGFR- and/or EGFRvIII-independent OSMR signaling. This may involve OSM–OSMR autocrine loops or OSMR complexes with other receptor tyrosine kinases. Studies exploring this possibility are well warranted.

This is the first study to show how the previously described EGFRvIII–STAT3 signaling pathway^12^ contributes to the tumorigenicity of HGG cells and to identify OSMR as a therapeutic target. It also further highlights the promiscuous interactions between EGFRvIII and resident receptors in HGG cells. Understanding how these interactions function is vital for developing novel, efficacious therapies for HGG, a highly lethal cancer.

## Figures and Tables

**Figure 1:**
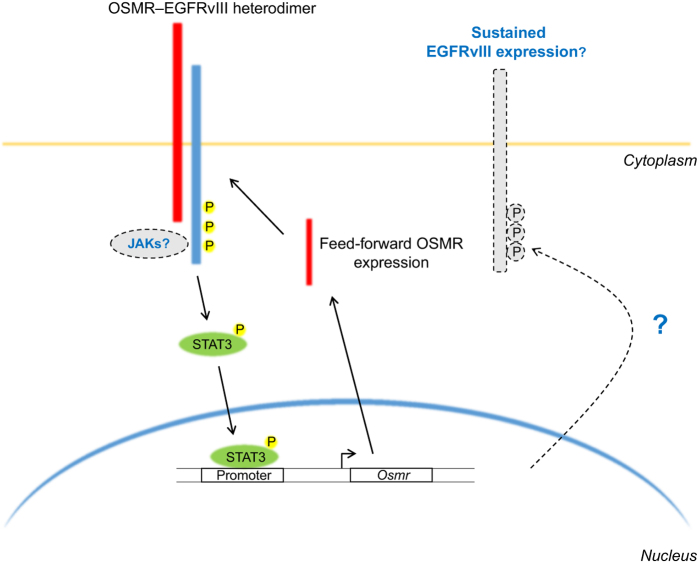
The EGFRvIII–OSMR heterodimer activates STAT3, initiating feed-forward expression of OSMR. EGFRvIII (blue) and OSMR (red) interact at the cell surface, leading to STAT3 (green) phosphorylation and phospho-STAT3 translocation to the nucleus. Nuclear phospho-STAT3 binds to the *Osmr* promoter and increases *Osmr* transcription. This results in feed-forward signaling through the EGFRvIII–OSMR heterodimer, which significantly increases HGG growth. Additional aspects of this model that could be explored in future are indicated by dashed lines. These include uncovering the role of JAK family kinases in activating STAT3 and determining whether phospho-STAT3 has a role in maintaining EGFRvIII expression. Yellow circles denote phospho-tyrosine.
